# Behavioral impact of national health campaigns on healthy lifestyle practices among young adults in Singapore: a cross-sectional study

**DOI:** 10.1186/s12889-021-11628-5

**Published:** 2021-08-30

**Authors:** Yong Zhi Khow, Talia Li Yin Lim, Jarret Shoon Phing Ng, Jiaxuan Wu, Chuen Seng Tan, Kee Seng Chia, Nan Luo, Wei Jie Seow

**Affiliations:** 1 Yong Loo Lin School of Medicine, National University of Singapore and National University Health System, 1E Kent Ridge Road, NUHS Tower Block Level 11, Singapore, 119228 Singapore; 2 Saw Swee Hock School of Public Health, National University of Singapore and National University Health System, 12 Science Drive 2, #10-01, Singapore, 117549 Singapore

**Keywords:** National health campaigns, Young adults, Lifestyle practices, Emerging adults, Life transitions

## Abstract

**Background:**

National health campaigns are often used to improve lifestyle behaviors in the general population. However, evidence specifically in the young adult population is scarce. Given the general deterioration of healthy lifestyle practices from adolescence to young adulthood, it is imperative to study this age group. This study aimed to investigate the behavioral impact of a national health campaign in Singapore on the lifestyle practices of young adults, and whether sex or full-time working and schooling status affected lifestyle practices.

**Methods:**

A total of 594 Singaporean respondents aged 18–39 years old were interviewed via a cross-sectional study in December 2019. Lifestyle practices assessed were diet, exercise, alcohol consumption, current tobacco use, and participation in health screening programs. Other factors investigated included exposure to the national health campaign “War on Diabetes” (WoD), sex, ethnicity, and working/schooling status. Multivariable modified Breslow-Cox proportional hazards models were used to estimate prevalence risk ratios (PRRs) as measures for the associations in this study, after adjusting for potential confounders.

**Results:**

Exposure to the WoD campaign had a significant association with meeting dietary recommendations (PRR = 1.6, 95% CI: 1.0–2.5, *p* = 0.037), participation in screening (PRR = 1.2, 95% CI: 1.0–1.5, *p* = 0.028), and current tobacco use (PRR = 0.5, 95% CI: 0.3–0.8, *p* = 0.003). Males were significantly more likely to meet exercise recommendations (PRR = 2.0, 95% CI: 1.5–2.7, *p* < 0.001), currently use tobacco (PRR = 3.9, 95% CI: 2.2–6.9, p < 0.001), and consume alcohol excessively (PRR = 1.5, 95% CI: 1.0–2.3, *p* = 0.046), as compared to females. Working young adults were significantly less likely to meet exercise recommendations (PRR = 0.7, 95% CI: 0.5–0.9, *p* = 0.019) but significantly more likely to be current tobacco users (PRR = 1.8, 95% CI: 1.1–3.1, *p* = 0.024), as compared to those who were in school.

**Conclusions:**

While this paper affirms that national health campaigns have significant beneficial associations in diet, health screenings and current tobacco use, policymakers should acknowledge that young adults are an age group with different influences that impact their healthy lifestyle habits. Specific interventions that target these subgroups may be required for better health outcomes. Future studies should evaluate other socio-environmental factors that could play a role in modifying the effect of health campaigns among young adults.

## Background

Based on the 2017 Global Burden of Disease Study, high blood pressure, high plasma glucose, and a high body mass index (BMI) were listed within the top 3 metabolic risk factors of premature mortality and morbidity [1]. Around the world, countries have adopted the use of national health campaigns to encourage healthy lifestyle practices, such as adopting a healthier diet [2], regular exercise [3], as well as reduced tobacco use [2] and alcohol consumption [4]. National health campaigns have been well-established to be an important component for getting the population to engage in healthy lifestyle behaviors [5, 6].

The largest national health campaign launched by Singapore’s Ministry of Health (MOH) in 2016, is known as the “War on Diabetes” (WoD) campaign [7]. This campaign was launched in light of the growing prevalence of Type 2 Diabetes Mellitus (DM), which is a chronic disease characterized by elevated blood glucose levels and relative insulin deficiency due to insulin resistance [8]. The worldwide burden of DM was 9.3% in 2019 [9]. In comparison, the prevalence rate of DM in Singapore was 14.2% in 2019 [10], and it is projected to increase to 15.6% of the population by 2050 [11], hence potentially having a large contribution to the disease burden in Singapore.

The WoD campaign comprised efforts to promote a healthy lifestyle amongst the general population in Singapore, which were aimed at various modifiable risk factors associated with DM including obesity [12], physical inactivity [13], unhealthy diet, tobacco use, and alcohol consumption [14]. Apart from policy and structural changes, the campaign also consisted of a publicity and educational component aimed at raising awareness of diabetes and emphasizing individual responsibility for one’s health [7]. The “Let’s Beat Diabetes” campaign, which was publicized through digital and out-of-home media platforms (e.g. bus stop shelters, taxis, public train screen doors) via advertisements and posters, was a public call-to-action for individuals to fight diabetes. Diabetes related roadshows with educational panels and talks on diabetes prevention were also conducted to give information on the constituents of a healthy diet and ethnic-specific healthy recipes and encourage exercise. The Health Promotion Board (HPB), under the MOH, utilized the HealthHub website to provide information regarding the harmful effects of alcohol consumption [15] and tobacco use [16–18]. A 5-question Diabetes Risk Assessment (DRA) tool [7] was also developed for those aged 18 to 39 years old to develop a greater awareness of their future risk of having DM.

After launching the various WoD campaign initiatives, there were early reports that the campaign was having a positive impact on the healthy lifestyle practices of the general public [19]. These reports made reference to the National Steps Challenge launched in 2015, with the objective of promoting an active lifestyle through increasing physical activity, which saw a 5-fold increase in participants in 2018, with over 800,000 Singapore residents participating in the challenge [20]. These reports also made reference to the National Nutrition Survey in 2018, which found that Singapore residents’ diet quality had improved, evident from their increased intake in wholegrain, fruits and vegetables, and decreased average daily energy intake [21]. A recent study that investigated the impact of the WoD campaign among the Singapore population aged 30 to 64 years old, also found significant improvements in diet quality [22]. While this is encouraging news for the WoD campaign, we note that most of the aforementioned evidence attributed improvements to the campaign without considering the population’s exposure to the campaign [22, 23], making them ecological in nature [19]. 

Moreover, the aforementioned evidence was based on a general population across all ages. Yet, healthy lifestyle behaviors have been described to decline around the age of young adulthood [24]. The young adult population is unique in that they face important life transitions, such as developing a self-identity [25], and the transition from schooling to working, which involves taking up adult responsibilities [26–28]. This has been described to be a critical developmental period where various psychosocial, social and environmental variables are at play [29]. Previous studies have demonstrated how differences in sex place individuals under different pressures [30–34], such as mandatory military conscription for males which can have an impact on an individual’s lifestyle practices [33, 35]. Young adulthood is also the period where people are exploring new behaviors, and hence health interventions targeting young adults may promote long-term adoption of healthy behavior patterns later in life. In addition, while most unhealthy lifestyle practices such as alcohol and tobacco use are adopted in young adulthood [24, 36], these practices are preventable [37]. Targeting young adults has great potential for primary prevention strategies to inculcate life-long healthy habits in subsequent generations. However, it remains unclear how receptive young adults are to change after being exposed to national health campaigns during this critical period of development. This prompted us to carry out this study in order to evaluate exposure to the campaign, as well as to investigate the behavioral impacts of exposure to this national health campaign on individual healthy lifestyle practices specifically among young adults.

Given the paucity of studies that capture individuals in young adulthood [25, 38–40], this study sought to evaluate the behavioral impact of a national health campaign on the lifestyle practices of young adults aged 18 to 39 years old. Secondarily, we aimed to investigate if sex, and full-time working or schooling status, were important factors associated with healthy lifestyle practices.

## Methods

### Study population

A total of 665 participants were recruited via convenience sampling from 16 to 21 December 2019. The participants were interviewed using an interviewer-administered, validated survey on healthy lifestyle practices. All participants recruited in this cross-sectional, quantitative study were Singapore Citizens or Permanent Residents aged 18 to 39 years old. Young adults are defined as those between the ages of 18–39 [41–48], as this age group captures a broad developmental period [46] prior to the onset of routine clinical interventions for all individuals at age 40 onwards [12, 49]. We excluded participants who were pre-diabetic or had either Type 1 or Type 2 DM from recruitment, as these individuals would often have regular medical follow-ups that would have affected their lifestyle practices. Participants who were concurrently schooling and working (*n* = 44), as well as participants who were neither schooling nor working at the time of survey (*n* = 27), were excluded from the data analysis step, in order to evaluate the impact of full-time working versus full-time schooling on healthy lifestyle practices. This leaves a total of 594 participants with complete information for the data analysis. This study was approved by the National University of Singapore’s Institutional Review Board (S-19-346).

### Data collection

Participants were recruited from a total of 23 different areas island-wide, from a mix of business districts (*n* = 9), tertiary institutions (*n* = 9), and housing estates (*n* = 5). Interviewers approached young adults encountered in the recruitment locations to check if they were willing to participate in our survey and if they fulfilled our inclusion criteria. Informed consent was collected from all our participants prior to participation. Verbal consent was obtained from participants aged 21 years old and above, while verbal parental consent was obtained from participants aged below 21 years old. Upon collecting informed consent and ensuring that our participants fulfilled our inclusion criteria, interviewers proceeded to read out the survey and captured the responses using their mobile devices.

### Survey

All participants were asked for their demographic factors such as age, sex, ethnicity, housing type, highest educational qualification, and whether they are working or schooling. Schooling participants are attendants of full-time formal education, including but not limited to education at universities, polytechnics, or any Institute of Technical Education. We determined each participant’s exposure to the WoD campaign by asking them whether they have “seen advertisements from the WoD campaign”. Questions on lifestyle practices were taken from the Simple Lifestyle Indicator Questionnaire [50] comprising: diet, exercise, alcohol consumption, and current tobacco use. In addition, we included a question on whether participants “have used the DRA tool”, which we used as a proxy to assess participants’ participation in specific health screening services.

We dichotomized the participant’s responses into healthy or unhealthy lifestyle practices based on Singapore and international recommendations. For diet, participants who consumed at least 2 servings of fruits and vegetables per day [51, 52], and at least 2 servings of whole-grains per day [53, 54], were considered to have a healthy diet. For exercise, the Singapore HPB and WHO recommends at least 150 min of moderate intensity exercise or at least 75 min of vigorous intensity exercise per week [55, 56]. Participants who had moderate intensity exercise of at least 4 times per week or vigorous intensity exercise of at least 3 times per week with a minimum of 30 min duration in each session, were considered to have a healthy lifestyle practice. For alcohol consumption, excessive alcohol consumption was defined as more than 1 drink for females and 2 drinks for males in a day as recommended by the Centers for Disease Control and Prevention [57]. Excessive alcohol consumption was considered an unhealthy lifestyle practice. Participants who responded “yes” to current tobacco use, were also considered as having an unhealthy lifestyle practice. Lastly, participants who responded “yes” to having used the DRA tool before, were considered as having participated in screening which constitutes a healthy lifestyle practice.

### Statistical analysis

Descriptive statistics consist of counts and percentages for categorical variables, and means and standard deviations for continuous variables. As our outcome measures are common activities, the odds ratio is not a good approximation for the prevalence risk ratio (PRR). Therefore, to estimate the independent effect of WoD campaign and other independent factors on each lifestyle practice, adjusted PRR for each factor was obtained using a multivariable modified Breslow-Cox proportional hazards model [58]. Exposure to WoD campaign, sex, working/schooling status, and ethnicity were included into the multivariable model for each lifestyle practice. Age and highest educational qualification were excluded from the multivariable models as these variables have high collinearity with working/schooling status. Housing type was also excluded from the multivariable models due to fundamental discrepancies between younger participants who lived with their parents and older participants who owned their own home.

The minimum sample size based on the Singapore population of 5.6 million, assuming a maximum variability of 50%, with a medium effect size of 0.5 [59, 60], with a ± 5% margin of error and 95% confidence level, was 385 complete responses.

All statistical analyses were done using RStudio version 1.2 and R Commander (Rcmdr: R Commander, R package version 2.6–2.). A *p*-value of less than 0.05 was deemed as statistically significant.

## Results

A total of 594 participants were analyzed in this study. Our participants were 25.5 ± 5.7 (range, 18 to 39) years old (Table 1), with a slightly larger proportion of them being 21–23 years old schooling participants (33.0%), so as to achieve equivalent sample sizes for comparison with working participants. We had a similar distribution of males (48.7%) and females (51.3%), as well as working (55.9%) and schooling (44.1%) participants. Majority of the participants were Chinese (81.0%), lived in public housing (71.7%), possessed an education qualification below a university degree (58.2%), and were exposed to the WoD campaign (60.9%). Overall, the majority of participants met exercise recommendations (67.7%), but only a minority of participants met dietary recommendations (17.7%) and have used the health screening tool (21.5%) (Table 2). Only a minority of the participants consumed alcohol excessively (14.5%) and were current smokers (11.3%).

**Table 1:** Summary of participants’ demographics

Participants (*n* = 594)	Summary statistics: n (%)
Age; mean ± SD	25.5 ± 5.7
Sex	
Males	289 (48.7)
Females	305 (51.3)
Working/Schooling Status	
Working	332 (55.9)
Schooling	262 (44.1)
Highest Education Qualification	
Below University Degree	346 (58.2)
University Degree and higher	248 (41.8)
Ethnicity	
Chinese	481 (81.0)
Non-Chinese	113 (19.0)
Housing Type	
Public Housing	426 (71.7)
Private	168 (28.3)
WoD campaign	
Exposed	362 (60.9)
Not Exposed	232 (39.1)

*SD* standard deviation, *WoD* “War on Diabetes”

**Table 2:** Prevalence of outcomes in each lifestyle domain

	Prevalence: n (%)		
Lifestyle domain	Overall	Exposed to WoD	Unexposed to WoD
Healthy Diet	105 (17.7)	75 (12.6)	30 (5.1)
Healthy Exercise	402 (67.7)	250 (42.1)	152 (25.6)
Excessive Alcohol Consumption	86 (14.5)	45 (7.6)	41 (6.9)
Current Tobacco User	67 (11.3)	27 (4.6)	40 (6.7)
Participated in Screening	128 (21.5)	90 (15.1)	38 (6.4)

*WoD* “War on Diabetes”

### Diet, exercise, and screening

The proportion of people who met the minimum dietary recommendations was significantly higher among those who were exposed to the WoD campaign as compared to the unexposed (adjusted PRR = 1.6, 95% CI: 1.0–2.5, *p* = 0.037) (Table 3). Exposure to the WoD campaign was not significantly associated with meeting minimum exercise recommendations. However, sex was a significant factor where the proportion of people who met the minimum exercise recommendations among males was two times as high as females (adjusted PRR = 2.0, 95% CI: 1.5–2.7, *p* < 0.001). Working status was the other significant factor where lower proportion of working individuals met the minimum exercise recommendations as compared to schooling individuals (adjusted PRR = 0.7, 95% CI: 0.5–0.9, *p* = 0.019). Exposure to the WoD campaign also had a significant positive association with participation in health screening (adjusted PRR = 1.2, 95% CI: 1.0–1.5, *p* = 0.028). The remaining factors were not significantly associated with screening participation.

**Table 3:** Prevalence risk ratios (PRRs) from univariable and multivariable analysis by using modified Breslow-Cox proportional hazards model

Outcome	Univariable analysis		Multivariable analysis	
	PRR (95% CI)	*p*-value	Adjusted PRR (95% CI)	*p*-value
Healthy Diet				
Exposure to WoD campaign				
Unexposed	Ref.		Ref.	
Exposed	1.7 (1.1, 2.6)	**0.013**	1.6 (1.0, 2.5)	**0.037**
Sex				
Females	Ref.		Ref.	
Males	1.4 (1.0, 2.1)	0.062	1.3 (0.9, 1.9)	0.202
Working/Schooling Status				
Schooling	Ref.		Ref.	
Working	1.4 (0.9, 2.0)	0.119	1.4 (0.9, 2.0)	0.143
Ethnicity				
Chinese	Ref.		Ref.	
Non-Chinese	0.7 (0.5, 1.2)	0.185	0.8 (0.5, 1.3)	0.312
Healthy Exercise				
Exposure to WoD campaign				
Unexposed	Ref.		Ref.	
Exposed	1.1 (0.9, 1.5)	0.370	1.2 (0.9, 1.6)	0.179
Sex				
Females	Ref.		Ref.	
Male	2.0 (1.5, 2.7)	**< 0.001**	2.0 (1.5, 2.7)	**< 0.001**
Working/Schooling Status				
Schooling	Ref.		Ref.	
Working	0.6 (0.5, 0.9)	**0.004**	0.7 (0.5, 0.9)	**0.019**
Ethnicity				
Chinese	Ref.		Ref.	
Non-Chinese	1.3 (0.9, 1.9)	0.227	1.4 (1.0, 2.1)	0.081
Participation in Screening				
Exposure to WoD campaign				
Unexposed	Ref.		Ref.	
Exposed	1.2 (1.0, 1.5)	**0.024**	1.2 (1.0, 1.5)	**0.028**
Sex				
Females	Ref.		Ref.	
Male	0.9 (0.8, 1.1)	0.256	0.9 (0.8, 1.1)	0.391
Working/Schooling Status				
Schooling	Ref.		Ref.	
Working	1.1 (0.9, 1.3)	0.528	1.1 (0.9, 1.3)	0.560
Ethnicity				
Chinese	Ref.		Ref.	
Non-Chinese	1.0 (0.8, 1.2)	0.936	1.0 (0.8, 1.3)	0.991

*PRR* prevalence risk ratio, *CI* confidence interval, *Ref* reference, *WoD* War on Diabetes

### Alcohol and tobacco

All the four factors were significantly associated with current tobacco use (Table 4). The proportion of people who were currently using tobacco among those who were exposed to the WoD campaign was half as high as those who were unexposed (adjusted PRR = 0.50, 95% CI: 0.30–0.80, *p* = 0.003). The proportion of smokers among males was almost 4 times as high as females (adjusted PRR = 3.9, 95% CI: 2.2–6.9, *p* < 0.001). Working individuals were more likely to smoke as compared to schooling individuals (adjusted PRR = 1.8, 95% CI: 1.1–3.1, *p* = 0.024). Non-Chinese individuals were also more likely to smoke as compared to Chinese individuals (adjusted PRR = 2.8, 95% CI: 1.7–4.6, *p* < 0.001).

**Table 4:** Prevalence risk ratios (PRRs) from univariable and multivariable analysis by using modified Breslow-Cox proportional hazards model

Outcome	Univariable analysis		Multivariable analysis	
	PRR (95% CI)	*p*-value	Adjusted PRR (95% CI)	*p*-value
Excessive Alcohol Consumption				
Exposure to WoD campaign				
Unexposed	Ref.		Ref.	
Exposed	0.7 (0.4, 1.0)	0.088	0.7 (0.5, 1.1)	0.093
Sex				
Female	Ref.		Ref.	
Male	1.5 (1.0, 2.3)	**0.049**	1.5 (1.0, 2.3)	**0.046**
Working/Schooling Status				
Schooling	Ref.		Ref.	
Working	1.0 (0.7, 1.6)	0.827	1.1 (0.7, 1.8)	0.568
Ethnicity				
Chinese	Ref.		Ref.	
Non-Chinese	0.7 (0.4, 1.3)	0.324	0.7 (0.4, 1.3)	0.253
Current Tobacco User				
Exposure to WoD campaign				
Unexposed	Ref.		Ref.	
Exposed	0.4 (0.3, 0.7)	**< 0.001**	0.5 (0.3, 0.8)	**0.003**
Sex				
Female	Ref.		Ref.	
Male	3.6 (2.1, 6.3)	**< 0.001**	3.9 (2.2, 6.9)	**< 0.001**
Working/Schooling Status				
Schooling	Ref.		Ref.	
Working	1.8 (1.1, 3.0)	**0.028**	1.8 (1.1, 3.1)	**0.024**
Ethnicity				
Chinese	Ref.		Ref.	
Non-Chinese	3.1 (1.9, 5.1)	**< 0.001**	2.8 (1.7, 4.6)	**< 0.001**

*PRR* prevalence risk ratio, *CI* confidence interval, *Ref* reference, *WoD* War on Diabetes

Exposure to the WoD campaign was not significantly associated with excessive alcohol consumption. Only sex was a significant factor where the proportion of people who consume alcohol excessively was higher among males as compared to females (adjusted PRR = 1.5, 95% CI: 1.0–2.3, *p* = 0.046) (Table 4).

## Discussion

This study aimed to evaluate the effect of national health campaigns on lifestyle practices of young adults. Additionally, it aimed to identify other socio-demographic factors that may play an important role on the lifestyle practices of young adults. Only predictors that were found to be significantly associated with more than one lifestyle practice were considered to be important factors. This study found that the exposure to a national health campaign had significant positive associations with healthy practices in the areas of diet, screening and current tobacco non-use. Moreover, this study found that participants who are schooling had significantly better healthy practices in the areas of exercise and current tobacco non-use as compared to those who are working. We also found that males as compared to females, had a significant positive association with meeting the minimum physical activity guidelines, but also had significant positive association with unhealthy practices in terms of alcohol and current tobacco use.

### National health campaign

In this study, we found that exposure to a national health campaign, such as the WoD campaign, had a significant positive association with the lifestyle practices among young adults. Participants who were exposed to the national health campaign were more likely to meet dietary recommendations, participate in health screening, and not currently use tobacco. However, exposure to the national health campaign was not significantly associated with alcohol consumption. This pattern of influence in the young adult age group was consistent with a review in the general population, that found mass media campaigns to have moderate to strong evidence for benefit when it comes to nutrition, tobacco usage, and screening, but only little evidence for benefit when it comes to alcohol consumption [6]. Other studies conducted in the general population have similarly reported significant reduction in tobacco usage with the use of effective health campaigns [61–63], but only limited improvement when it comes to reducing alcohol consumption [64, 65]. Interestingly, even though alcohol and tobacco have been described to possess similar competing influences against positive behavior change (i.e. marketing, pricing, social norms and addiction) [6], reducing alcohol consumption seems to be more resistant to the effects of national health campaigns compared to tobacco use among young adults. This may be because alcohol is still considered a socially acceptable beverage in social settings such as gatherings with family and friends [66, 67]. This is in contrast to using tobacco, which is stigmatized to be a vice [68], and is fast becoming frowned upon in many professional settings [69]. As such, public health authorities can consider exploring other methods of portraying excessive alcohol consumption as an unhealthy lifestyle decision in the public consciousness. Nonetheless, these findings affirm the use of mass media campaigns to promote healthy nutrition, discourage tobacco use and encourage participation in screening programs.

Interestingly, this study found that exposure to the national health campaign was not significantly associated with meeting exercise recommendations. This was contrary to other studies that have demonstrated health campaigns to be effective in increasing physical activity among the general population [70–72]. Health campaigns have been described to have moderate evidence for benefit [6]. This might be because the overall prevalence of participants who already met exercise recommendations, regardless of their exposure to the national health campaign, was high. This may indicate that the majority of young adults are self-motivated in keeping active, or it may be the effect of mandatory military conscription in Singapore which has boosted the prevalence.

### Working and schooling

We also found that full-time working and schooling status had a significant association with the lifestyle practices of young adults. Working participants were less likely to meet the minimum exercise recommendations and more likely to currently use tobacco compared to the schooling population. Schooling participants have more unstructured time weaved into their curriculums and more opportunities to access organized sporting activities, such as collegiate athletics or clubs [73–75]. Whereas in the workplace, a lack of unrestricted time coupled with an unsuitable physical environment are the common barriers [76] that make working adults less likely to achieve the minimum exercise recommendations. Meanwhile, the financial independence that comes with taking on a full-time job may play a contributory role in higher rates of tobacco use among the working participants, because higher income has been found to reduce price sensitivity to cigarettes among tobacco users [77]. Financial independence also results in the autonomy to spend, as young working adults may see an elimination in parental restrictions on cigarettes bought with their own money rather than with their parents’ money [78]. Furthermore, moving from school to the workplace also meant no school-imposed sanctions on tobacco use [79, 80]. These environmental factors could explain the difference between lifestyle practices among schooling and working participants within this age group. Meanwhile, schools can consider moving beyond solely relying on disincentives to deter tobacco consumption and focus on the root cause by educating young adults on different methods to cope with stress situations while practicing financial prudence [81].

### Sex

In this study, sex had a significant association with the lifestyle practices of young adults. Specifically, males were more likely to meet exercise recommendations, but also more likely to currently use tobacco and consume alcohol excessively. In Singapore, all able-bodied males above the age of 18, have to serve a mandatory full-time conscription of 2 years, and continue to be operationally fit for duty until the age of 40 years old [82]. While females are also able to join the conscription on a volunteer basis, they make up the minority of service personnel in the Singapore armed forces. This may explain why males in this age group were more likely to meet the minimum exercise recommendations, regardless of their exposure status to the WoD campaign. However, military service has been associated with the adoption of some unhealthy lifestyle practices such as tobacco use and alcohol consumption. Due to the tight laws in Singapore, participants are likely to underreport and not attribute their adoption of these practices to their time in the armed forces [83]. However, a study on the Republic of Korea Air Force soldiers found that 24.6% of survey respondents reported picking up smoking after joining military service [35]. A thorough review into the American armed forces conducted by the Department of Defense [84], also revealed that individuals were more likely to pick up tobacco use and alcohol consumption in the military. As high as 38% of service personnel had picked up tobacco use after enlisting, and 5% claim that they smoked in order to “fit in with their military unit”. In addition, 55% of tobacco users reported that they smoke when consuming alcohol, suggesting the close relationship between both unhealthy lifestyle practices. Up to 82% and 93% of participants also reported using tobacco and consuming alcohol when around their friends in order to facilitate social networking. It is believed that such activities have a long tradition in the military and that it forges stronger camaraderie among fellow soldiers [85]. Different social pressures might be one of the unique factors in this age group to consider which may explain why sex was moderately associated with some lifestyle practices. Further investigation to elucidate the source of these social pressures may be important to institute appropriate interventions to target the male young adult population.

### Limitations

This study has several limitations. One limitation was the cross-sectional nature of this study. Therefore, we acknowledge our inability to establish causality in the relationships. Nonetheless, we have a low proportion of respondents who were excluded from final analysis, with a final analyzed proportion of 89% of our survey respondents. Another limitation was our use of a non-probability, convenience sampling method. This was due to the presence of strict confidentiality laws in Singapore which precludes our possession of a list of personal data for the systematic recruitment of our participants. However, we have made every effort to strive at a representative sample, by recruiting a large number of participants (total *n* = 665) throughout an array of different locations island-wide (*n* = 23). In addition, the age restriction of the sample population to young adults, may limit the generalizability of the findings to individuals of other age groups. However, this also constituted a strength of this study, as to the best of our knowledge, this is one of the first studies of its kind, to have directly investigated the associations between exposure to a national health campaign and various lifestyle practices young adults aged 18 to 39 years old. In doing so, this study has raised interesting findings regarding this age group, which should serve as the springboard for further evaluation in future studies. This age group, which is neither completely appreciated when subsumed under the label of adolescence nor adults, often experience critical transitions in multiple aspects of their life at a time, which are deserving of special attention in the field of public health and preventive medicine.

### Future studies

We acknowledge that there are other socio-environmental factors that may affect the impact of health campaigns on the young adult age group. These factors, detailed below, were not covered in our current study but are important and could be the topic of investigation in future studies. In particular, perceived health risks amongst young adults could play a role because younger people have been found to have a more optimistic perception of their health compared to older age cohorts [86–88]. As such, they might not prioritize adoption of healthy lifestyle practices regardless of exposure to the WoD campaign. Moreover, many young adults get married during this age group. Thus, we postulate that marriage plays a role in one’s lifestyle practices given that it has been described to be associated with body mass index and weight related behaviors, including exercise [89]. The emergence of parenthood is also a unique and interesting factor to investigate in this age group. Previous studies have revealed that new parenthood is also associated with lower physical activity levels amongst the decline of other healthy practices [90, 91]. The responsibilities and commitments of new parenthood may exhaust young adults to disregard healthy lifestyle practices [92], or it may lead to the adoption of healthy lifestyle practices so as to set an exemplary behavior for their children to follow [93]. While these factors are beyond the scope of this study, they deserve further evaluation in future studies to understand the unique circumstances young adults face, and their effect on the adoption/deterioration of certain lifestyle practices during this age.

Moreover, there are many new programs as part of the WoD campaign that are soon to be implemented in the community [94]. While this study showed encouraging early behavioral impact of the WoD campaign on individuals, change in overall outcomes takes time to manifest [95, 96]. It would be valuable for future studies to follow up on the subsequent initiatives and evaluate how they augment the campaign’s current outreach and impact.

## Conclusions

While this paper affirms that national health campaigns have significant beneficial associations in diet, health screenings and current tobacco use, policymakers should acknowledge that young adults are an age group with different influences that impact healthy lifestyle habits, such as working/schooling status. Specific interventions that target these subgroups may be required for better health outcomes. Future studies should evaluate other factors that may augment the effect of national health campaigns among young adults, such as career instability, military involvement, and new parenthood.

## Data Availability

The datasets used and/or analyzed during the current study are available from the corresponding author on reasonable request.

## Abbreviations

BMI: Body mass index
DM: Diabetes mellitus
DRA: Diabetes risk assessment
HPB: Health promotion board
MOH: Ministry of health
PRR: Prevalence risk ratio
WHO: World health organization
WoD: War on diabetes
